# Cardiac Tumors Revisited

**DOI:** 10.21470/1678-9741-2024-0450

**Published:** 2025-09-26

**Authors:** Khaled Ebrahim Al-Ebrahim

**Affiliations:** 1 King Abdulaziz University, Jeddah, Saudi Arabia. E-mail: dr.k.ebrahim@gmail.com

Dear Editor,

I read with interest the article “Cardiac Tumors: Review” by Karigyo et al.^[1]^ and I commend the authors for their excellent review. In 2013, I published a case report, which was oversighted by the reviewers, about a metastatic cardiac tumor from cancer cervix in a 22-year-old woman^[2]^. The peculiarity of this tumor is that it was metastasizing to the coronary sinus as the first report in literature. This patient was diagnosed one year after radical hysterectomy for stage IV B cervical squamous cell carcinoma, followed by radiation and chemotherapy, and presented with dyspnea and palpitations. Echocardiography showed a large right atrial mass, protruding across the tricuspid valve, resembling a myxoma. Intraoperatively, under cardiopulmonary bypass and cardioplegic arrest, the mass was found to be filling the right atrium. Excision of the mass revealed that its origin was the coronary sinus extending to the triangle of Koch. The histopathology of the mass and cytology of the pericardial fluid confirmed a metastatic squamous cell carcinoma of uterine origin. A repeat echocardiogram six months later showed no recurrence. The mechanism of spread was most likely hematogenous through the coronaries and residing in the coronary sinus.
